# Supplementary material for: Impact of Microbiota Depletion by Antibiotics on SARS-CoV-2 Infection of K18-hACE2 Mice
Source: Cells. 2022 Aug 18;11(16):2572. doi: 10.3390/cells11162572 (PMC9406363; doi:10.3390/cells11162572)
Supplement: Supplementary file 1 [file cells-11-02572-s001.zip › cells-1844143-supplementary.pdf]

Supplementary Materials

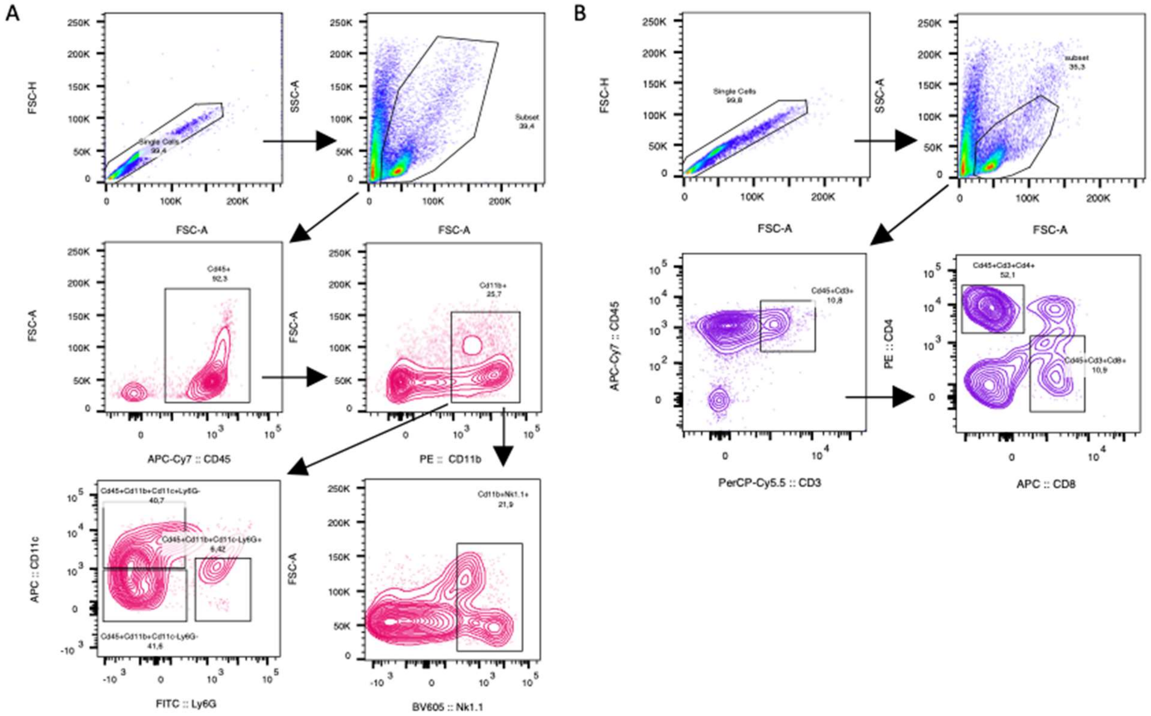

Supplementary Figure S1. Gating strategies used to define the leukocyte populations of the BAL. (A) Gating strategy used for analysis of myeloid and NK cells. (B) Gating strategy used for the analysis of lymphocytes.

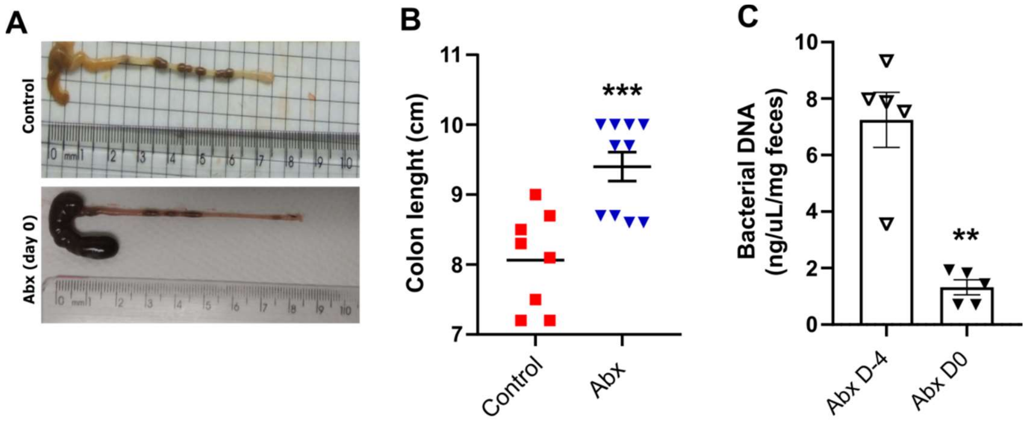

Supplementary Figure S2. Effect of oral antibiotics on the length of large intestine and fecal bacterial load. (A) Representative images of cecum and colon. Female K18-hACE2 mice were treated for 3 days with antibiotic cocktail before infection (Abx day 0). (B) Colon length of mice treated or not with antibiotics. ***p<0.001 by Student's t-test (n= 8-11). (C) Bacterial DNA from feces samples of mice before (Abx D-4) and after 3 days of antibiotic treatment (Abx D0). **p<0.005 by Student's t-test (n=5).

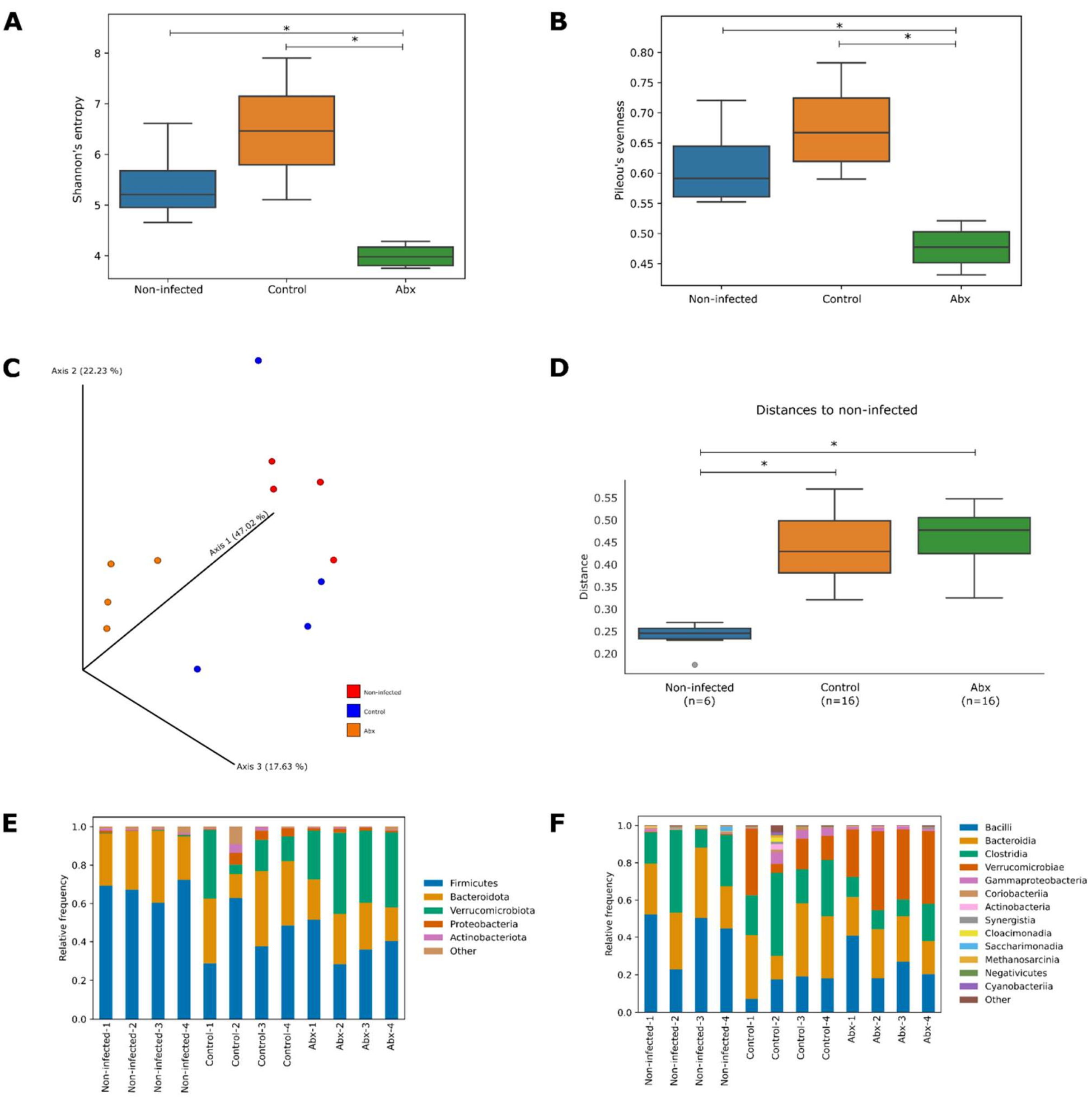

Supplementary Figure S3. Microbiota changes after infection of K18-hACE2 mice. Female K18-hACE2 mice were either treated (Abx) or not treated (Control) for 3 days before SARS-CoV-2 infection. Mice were euthanized at 5 dpi and the luminal contents were collected for microbiota analysis. A non-infected group was also included in this analysis. Comparisons of alpha diversity between experimental groups. (A) Shannon's entropy index, (B) Pielou's evenness index. *p<0.05 by Kruskal–Wallis test (n= 4 samples/group). (C) PCoA plot using weighted UniFrac dissimilarity based on ASVs of the different experimental groups. Comparisons of beta diversity between experimental groups. (D) Distance from the non-infected group using pairwise PERMANOVA test and weighted UniFrac distance matrix. *p<0.05 (n= 4 samples/group). (E-F) Comparison of gut bacterial composition among experimental groups. (E) Bacterial abundance at the phylum level and (F) class distribution.
